# Development of a Functionally Minimized Mutant of the R3C Ligase Ribozyme Offers Insight into the Plausibility of the RNA World Hypothesis

**DOI:** 10.3390/biology3030452

**Published:** 2014-07-29

**Authors:** Eri Kurihara, Sayuri Uchida, Takuya Umehara, Koji Tamura

**Affiliations:** 1 Department of Biological Science and Technology, Tokyo University of Science, 6-3-1 Niijuku, Katsushika-ku, Tokyo 125-8585, Japan; E-Mails: kurieri123@yahoo.co.jp (E.K.); j8313611@ed.tus.ac.jp (S.U.); tumehara@rs.noda.tus.ac.jp (T.U.); 2 Research Institute for Science and Technology, Tokyo University of Science, 2641 Yamazaki, Noda, Chiba 278-8510, Japan

**Keywords:** R3C ligase ribozyme, minimization, RNA world, origin of life

## Abstract

The R3C ligase ribozyme is an artificial ligase ribozyme produced by modification of the ribozyme that lacks cytidine. Here, we attempted to modify the original R3C ribozyme (73 nucleotides) by reducing the number of nucleotides while maintaining the maximum possible catalytic efficiency. By partially deleting both the “grip” (P4 + P5) and “hammer” (P3) stem-loops, we found the critical border to retain activity comparable to that of full-length R3C. The three-way junction structure was necessary to maintain enzymatic function and the stability of the “grip” (P4 + P5) stem had a large influence on the catalytic activity of R3C. The final minimized ribozyme we obtained comprised ~50 nucleotides, comparable to the estimated length of prebiotically synthesized RNA. Our findings suggest that the autocatalytic function in ribozymes is indeed possible to obtain using sequence lengths achievable with prebiotic synthesis.

## 1. Introduction

Modern biology separates the roles of genetic information infrastructure and catalysis. The central dogma of biology holds that DNA and RNA are responsible for the former, and proteins for the latter, and that the genetic information transmits in the order of DNA→RNA→protein [1]. DNA is synthesized by using enzymes, and the sequences for these proteins are coded by the DNA. Therefore, the so-called chicken or the egg problem occurs, in which we cannot determine the order in which this paradigm arose, as both aspects are dependent on each other. The discovery of ribozymes [2,3] gave rise to the “RNA world” hypothesis [4], wherein the roles of information infrastructure and catalysis are postulated to have been performed by RNA alone.

For a self-sustaining RNA world to exist, the putative archaeo-RNA must be self-ligated. The presence of homochirality in biological systems provides support to this hypothesis. Previous work on the homochirality of nucleotides shows that homochirality itself could be crucial for ligation [5,6]. Other work has established the possibility that RNA homochirality may have brought about amino acid homochirality [7,8,9,10,11,12]. Although the above-referenced work supports the hypothesis, it is necessary to show that autocatalytic (self-replicating) RNA ligases are chemically feasible. To this end, researchers have sought to recreate them from extant RNA ribozymes. Using *in vitro* selection techniques [13], several synthetic RNA ligase ribozymes have been produced thus far. Some examples of these include class I ligase ribozyme [14], L1 ligase ribozyme [15], and R3C ligase ribozyme [16]. The class I ligase ribozyme is composed of 119 nucleotides (nt) and has the most productive ligation ability (~100 min^−1^) [14].

The crystal structure of the catalytic core of an RNA-polymerase ribozyme has been solved, showing a tripod scaffold through minor-groove interactions [17,18]. The original L1 ligase ribozyme comprising 130 nt was also isolated from randomized RNA [15], and it is one of the five ligase ribozymes known to catalyze the 5'–3' phosphodiester bond formation [15,19,20,21,22]. Minimum functional derivatives of L1 ribozymes have been shown to be as small as 56 nt long [23]. The crystal structure of L1 ligase ribozyme elucidated that the invariant residues stabilize a flexible stem of the ribozyme through tertiary interactions [24].

In the process of the formation of the RNA world, the length of an RNA sequence would be very important. Eigen insisted that the length of the nucleotides allowing accurate replication under Darwinian selection in enzyme-free nucleotide polymerization is no more than about 100 nt when the physical properties inherent to nucleic acids are considered [25]. RNA sequences synthesized in a clay mineral (montmorillonite) environment have been shown to be at most ~50 nt long [26]. These severe length restrictions highlight the scientific interest of the R3C ribozyme.

The R3C ligase ribozyme was originally derived from the *in vitro* selected ribozyme that lacks cytidine [20]. The R3C ribozyme shows slower ligation activity (0.32 min^−1^) as compared with that of class I ligase. However, the R3C ribozyme is much smaller than class I ligase (73 nt) [16]. In R3C, a three-way junction structure is formed around three stems (P2, P3, P4). Furthermore, it has been proven that the *trans*-version of R3C is capable of self-replication [27]. Although a rough estimate of the domains of R3C ligase ribozyme required for activity has been made [16], and its behavior as a self-replicating ribozyme have been investigated [27,28], to our knowledge, the precise minimal regions of R3C required for ligase activity have not been elucidated yet. Here, we present our efforts to find the minimal functional length of R3C. We prepared a number of nt-deleted mutants focusing on regions known to be crucial to catalytic activity. The ligase activities of the mutants were measured using the assay method of Rogers and Joyce [16], and studies of the structures of R3C and its mutants were performed using circular dichroism (CD) and computational simulation. We discuss our results in an evolutionary context.

## 2. Experimental

### 2.1. Plasmid Construction and in Vitro Transcription

Unlabeled deoxyribonucleotides were synthesized by Operon Biotechnologies Inc. (Tokyo, Japan). HPLC-purified 5'-terminal fluorescein- or 6-carboxyfluorescein (6-FAM)-labeled oligonucleotides were prepared by Japan Bio Services Co., Ltd. (Saitama, Japan). Synthetic DNA carrying the T7 promoter and the sequences corresponding to those of R3C ligase ribozyme, R3C-w/o-Hammer, R3C-w/o-Grip was ligated into the pTAC-1 vector using the TA PCR Cloning kit (BioDynamics Laboratory Inc., Tokyo, Japan), and transformed into *E. coli* strain DH5α. The template DNA sequences were confirmed by Operon Biotechnologies Inc. (Tokyo, Japan).

Each template DNA was prepared from these plasmids or chemically synthesized deoxyribonucleotides carrying the T7 promoter and the sequences corresponding to variants of R3C ligase ribozyme, and two synthetic primers using the polymerase chain reaction. RNA transcription was performed at 37 °C for 16 h in a reaction mixture containing 40 mM Tris-HCl (pH 8.0), 10 mM dithiothreitol, 2 mM spermidine, 8 mM MgCl_2_, 2.5 mM each NTP, template DNA (0.2 mg/mL), and pure T7 RNA polymerase (~100 µg/mL) [29]. The transcripts were purified by denaturing 12% polyacrylamide gel electrophoresis. The concentrations of purified obtained RNA were determined from the UV absorbance at a wavelength of 260 nm by using Implen NanoPhotometer (München, Germany).

### 2.2. Analysis of Ligation

Ligation analysis was performed by the method of Rogers and Joyce with a slight modification [16]. R3C ligase ribozyme or its variants dissolved in solution containing 50 mM Tris-HCl (pH 8.5) and 15 mM MgCl_2_ were first heated to 37 °C for 5 min and then cooled to 4 °C. Then, the ligation reaction was started by adding 1.5 µL of 50 µM substrate to the solution. The final concentration of the ribozyme and the substrate was 5 µM each. The volume of the reaction mixture was 15 µL. After incubation at 24 °C for 18.5 h, the solution was applied to denaturing 12% polyacrylamide gel for electrophoresis. The gel was analyzed on a Typhoon FLA 7000 (GE Healthcare Japan, Tokyo, Japan), and the ligated products were quantified by using Image Quant TL software. Although Rogers and Joyce used a DNA/RNA chimera substrate (5'-dCdGdAdCdTdCdAdCUAUA-3') in the original experiment [16], we used both RNA substrate (5'-CGACUCACUAUA-3') and DNA substrate (5'-dCdGdAdCdTdCdAdCdTdAdTdA-3').

### 2.3. CD Spectroscopy

The CD spectra [30] of the R3C ligase ribozyme and its mutants dissolved in Ultrapure water (Invitrogen) at room temperature were taken using a J-805 Circular Dichroism Spectrometer (JASCO Corporation, Tokyo, Japan). The cell path length was 1 mm with a 50 nm/min scanning speed. RNA concentrations were 20 µM for each sample.

### 2.4. Prediction of RNA Secondary Structures 

RNA secondary structures were predicted using the CentroidFold web server (provided by CBRC, AIST, Japan) [31,32,33]. The McCaskill model was used, and the gamma value was 16.

## 3. Results

### 3.1. Ligation Activities of R3C Ribozymes and of “Hammer”- or “Grip”-Completely Deleted Mutant

**Figure 1:**
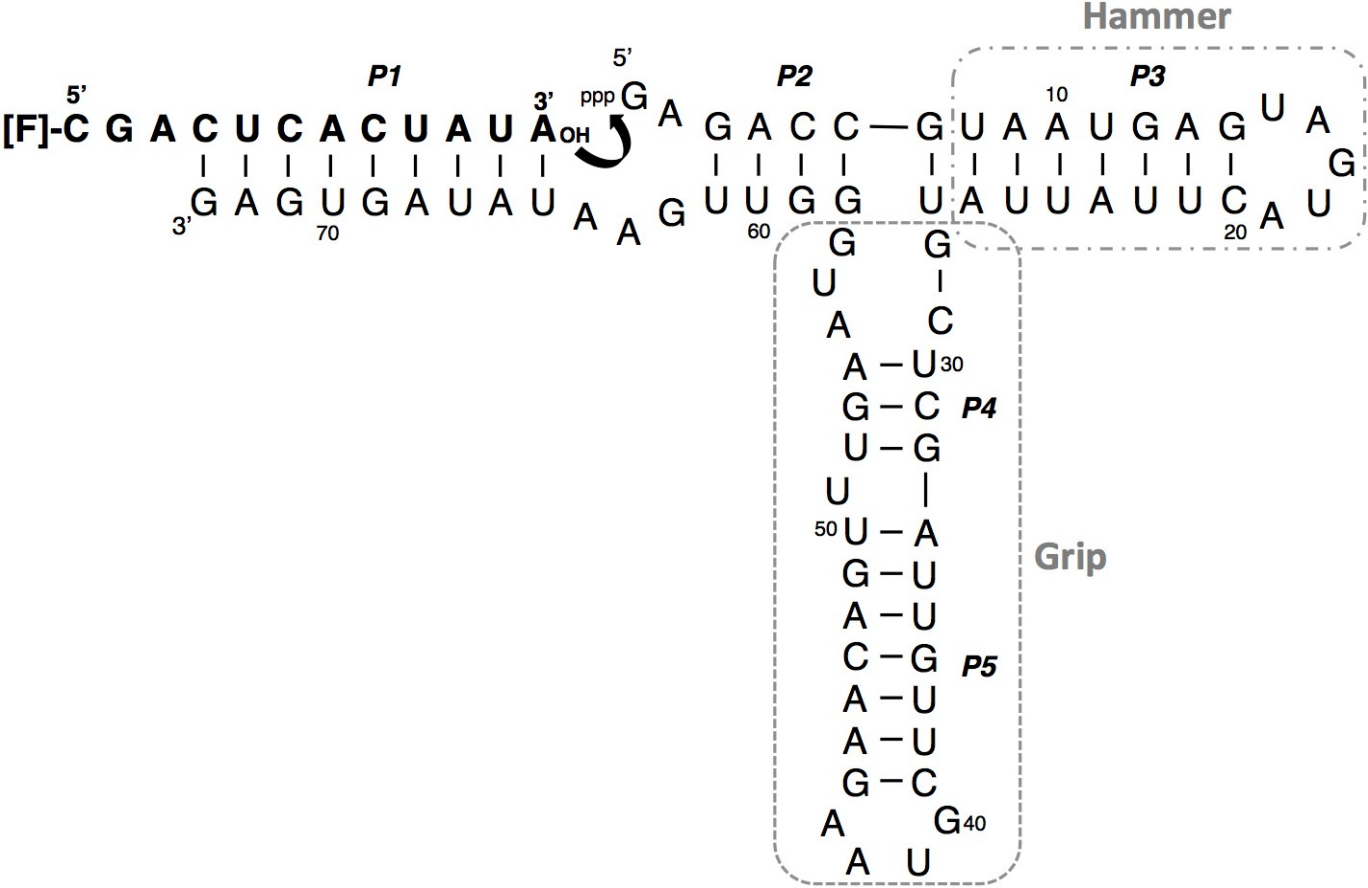
Composition of the R3C ligase ribozyme with fluorescence-labeled RNA substrate. The ribozyme is composed of 73 nucleotides and forms 5 paired regions (P1–P5). The stem-loop region contained P3, and the regions comprising P4 and P5 are newly designated as “hammer” and “grip”, respectively. The numbering of the nucleotides starts from the guanosine triphosphate at the 5'-end.

R3C ligase ribozyme (73 nt) was originally developed using *in vitro* selection from 74-nt R3 ribozyme [20]. R3C contains five paired regions (known as P1–P5). Its substrate hybridizes to the 3'-single-stranded region of the ribozyme and eventually forms P1 (Figure 1).

**Figure 2:**
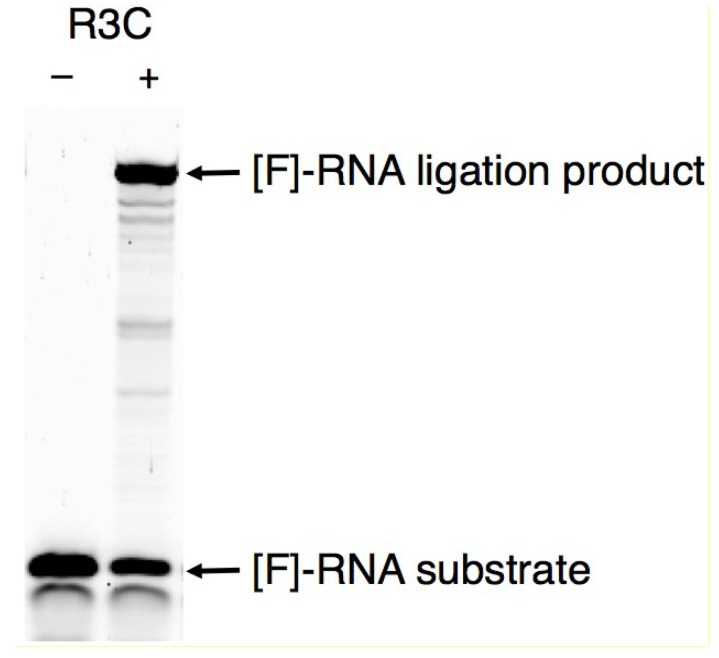
Analysis of the ligation product using fluorescence-labeled RNA substrate (5'-CGACUCACUAUA-3') with (+) or without (–) R3C ribozyme. After incubation at 24 °C for 18.5 h, the solution was applied to denaturing 12% polyacrylamide gel electrophoresis. The gel was analyzed on Typhoon FLA 7000 (GE Healthcare Japan, Tokyo, Japan), and the ligated products were quantified by using Image Quant TL software.

**Figure 3:**
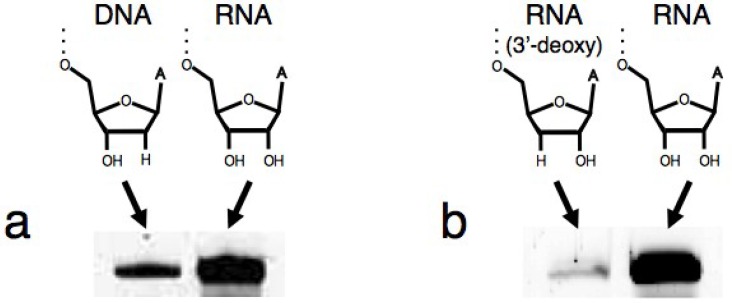
Analysis of the ligation product by R3C ribozyme. (**a**) DNA substrate (5'-dCdGdAdCdTdCdAdCdTdAdTdA-3'); (**b**) RNA substrate possessing 3'-deoxy adenosine at the 3'-terminal (5'-CGACUCACUAUA(3'-deoxy)-3'). The quantification methods are the same as in Figure 2.

Instead of using the original DNA/RNA chimera substrate, we used a RNA substrate to see the behavior of the ribozyme in “RNA” world. The quantity of the ligated products was measured after incubation at 24 °C for 18.5 h (Figure 2). At this time, the reactions reached plateau levels and the global tendency of the ribozyme could be determined. DNA substrate was also used in comparison. (DNA-RNA hybrid double helices are expected to exist as A-form double helices, similar to RNA-RNA duplexes [34].) In the case of the DNA substrate, the amount of the ligated product was 9.3% of that in RNA substrate (Figure 3a). In order to specify the ligation site, we also used the RNA substrate possessing 3'-deoxy adenosine at the 3'-terminal. The ligation activity was greatly decreased (3.7%) (Figure 3b), confirming the conclusion that the R3C ribozyme produces 5'–3' linkage [16].

**Figure 4:**
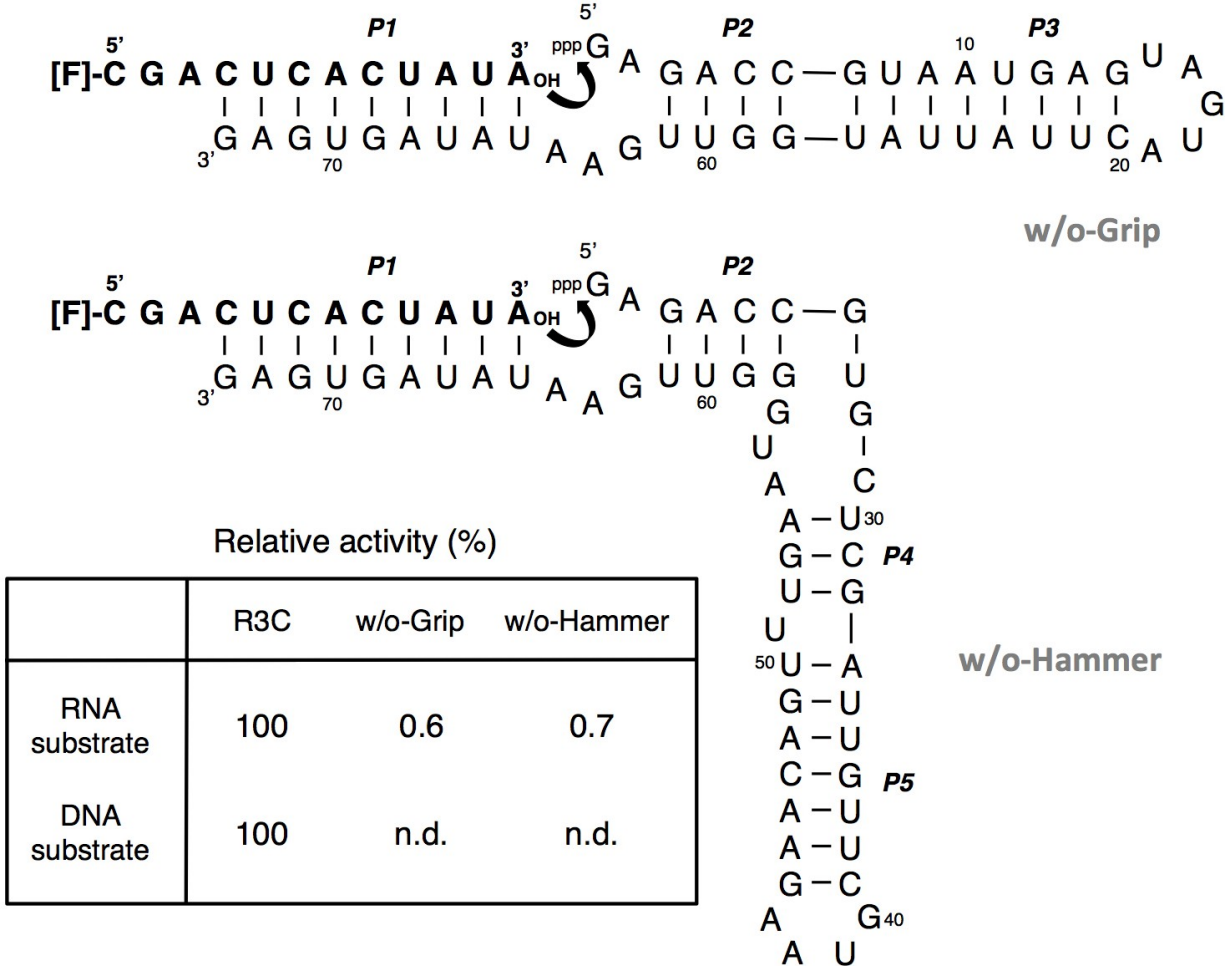
Ligation activities of R3C hammer- or grip-completely deleted mutants. The activities are shown as relative values (%) for both RNA and DNA substrates compared to those in the case of the full-length R3C ribozyme (100%). n.d. denotes not detected.

We then tried to construct a smaller-sized R3C ligase ribozyme that retains sufficient activity. We designate the stem-loop region composed of P3 as “hammer” and the region composed of P4 and P5 as “grip.” The two variants without hammer (R3C-w/o-Hammer) and without grip (R3C-w/o-Grip) displayed drastically decreased ligation activities in the presence of either RNA or DNA substrate. Even in the case of RNA substrate, the activities were <1% compared to that of the full-length R3C ligase ribozyme (Figure 4).

### 3.2. Ligation Activities of R3C “Grip”-Partially Deleted Mutants

**Figure 5:**
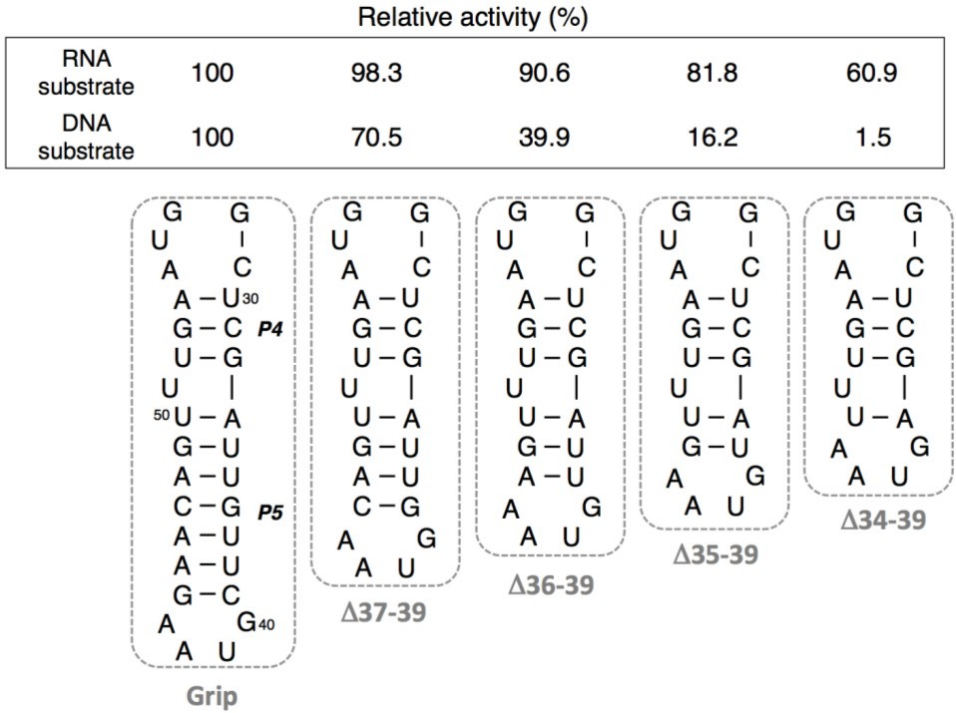
Ligation activities of R3C grip-partially deleted mutants. The activities were shown as relative values (%) for both RNA and DNA substrates compared to those in the case of the full-length R3C ribozyme (100%).

As R3C-w/o-Grip showed little ligation activity, we investigated the activities of R3C grip-partially deleted mutants. Compared to the full-length R3C ligase ribozyme, <Δ37-39>, which was a 3-bp deletion mutant from the end of grip stem (P5), indicated 98.3% (RNA substrate) and 70.5% (DNA substrate) activity after incubation at 24 °C for 18.5 h (Figure 5). Stepwise deletions of the single base pair of the stem from <Δ37-39> produced incremental decreases in the activities. <Δ36-39> and <Δ35-39> had 90.6% and 81.8% ligation activity of R3C ribozyme for RNA substrate, respectively, with 39.9% and 16.2% ligation activity for DNA substrate, respectively (Figure 5). One more deletion of the stem, <Δ34-39>, gave rise to a further decrease in the activities (60.9% for RNA substrate and 1.5% for DNA substrate, respectively) (Figure 5).

### 3.3. Ligation Activities of R3C “Hammer”-Partially Deleted Mutants

**Figure 6:**
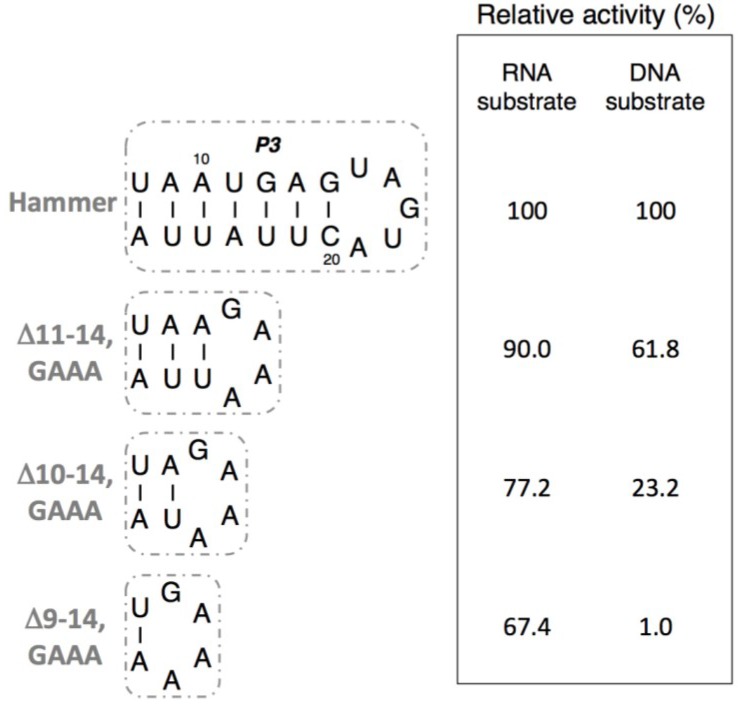
Ligation activities of R3C hammer-partially deleted mutants. The activities are shown as relative values (%) for both RNA and DNA substrates compared to those in the case of the full-length R3C ribozyme (100%).

We similarly studied the ligation activities of R3C hammer-partially deleted mutants. These mutants were made by deleting base pairs from the P3 stem and substituting the loop with the GAAA tetra loop. The 4-bp deletion mutant <Δ11-14,GAAA> still retained 90.0% activity for RNA substrate and 61.8% activity for DNA substrate, compared to those of the full-length R3C ribozyme (Figure 6). Subsequent deletions of base pairs produced further decline in activity, such that the <Δ10-14,GAAA> mutant displayed 77.2% activity for RNA substrate and 23.2% activity for DNA substrate, and the <Δ9-14,GAAA> mutant displayed 67.4% activity for RNA substrate and 1.0% activity for DNA substrate (Figure 6).

### 3.4. Ligation Activities of R3C “Grip” and “Hammer”-Double Partially Deleted Mutants

**Figure 7:**
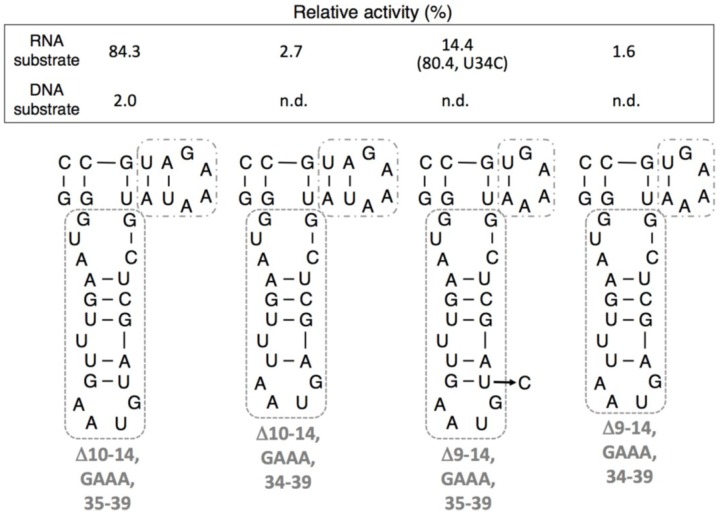
Ligation activities of R3C grip and hammer-double partially deleted mutants. The activities are shown as relative values (%) for both RNA and DNA substrates compared to those in the case of the full-length R3C ribozyme (100%). The ligation activity in the case of U34C mutant of <Δ9-14,GAAA,35-39> (<Δ9-14, GAAA,35-39,U34C>) is shown in parentheses. n.d. denotes not detected.

With the results of grip- or hammer-partially deleted mutants in mind, we then prepared grip- and hammer-deleted mutants. Among these mutants prepared, only <Δ10-14,GAAA,35-39> showed detectable activity in our experimental condition for DNA substrate. For RNA substrate, <Δ10-14,GAAA,35-39> displayed relatively high activity (84.3%). Other deletions decreased the ligation activities significantly (*i.e.*, <Δ10-14,GAAA,34-39> (2.7%), <Δ9-14,GAAA,35-39> (14.4%), and <Δ9-14,GAAA,34-39> (1.6%)) (Figure 7).

To investigate the role of the conceivable wobble base pair U34G49, we prepared the <Δ9-14,GAAA,35-39,U34C> mutant and measured the ligation activity for RNA substrate. Although the size of this mutant is the same as <Δ9-14,GAAA,35-39>, the activity increased drastically (80.4%), comparable to that of <Δ10-14,GAAA,35-39> (Figure 7).

### 3.5. CD Spectra of the Ligase Ribozymes

**Figure 8:**
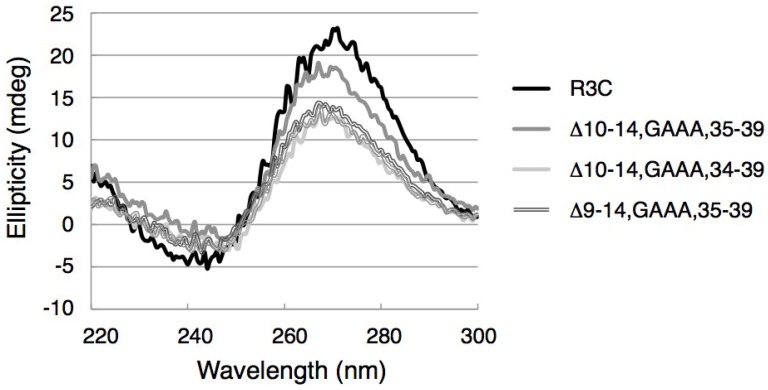
CD spectra of the R3C ribozyme and R3C grip and hammer-double partially deleted mutants. The measurements were performed at room temperature.

The CD spectra of the full-length R3C ligase ribozyme and of R3C grip and hammer double partially deleted mutants were measured. The R3C ligase ribozyme showed a positive Cotton effect at around 270 nm and a negative Cotton effect at around 244 nm (Figure 8). In the cases of double partially deleted mutants, the spectra were similar but not the same. The positive CD band at around 270 nm was shifted toward shorter wavelengths, and the negative CD band at around 244 nm was shifted toward longer wavelengths (Figure 8).

### 3.6. Secondary Structure Prediction of the Ligase Ribozymes

**Figure 9:**
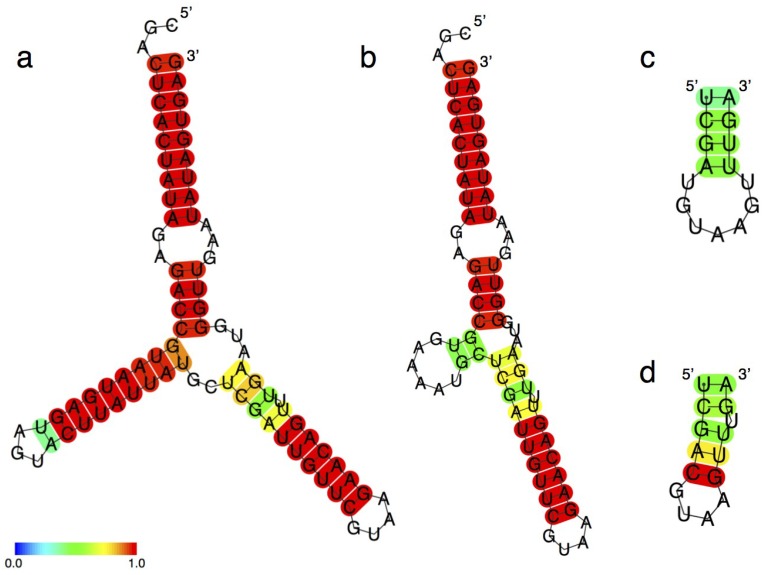
Secondary structure prediction of the ligase ribozymes using CentroidFold web server [33]. (**a**) Ligation product of full-length R3C ribozyme; (**b**) Ligation product of hammer-partially deleted mutant <Δ9-14,GAAA>; (**c**) Regions P4 and P5 in <Δ9-14,GAAA,35-39>; (**d**) Regions P4 and P5 in <Δ9-14,GAAA,35-39,U34C>. The McCaskill model was used, and the gamma value was 16. Each predicted base pair is colored with the heat color gradation from blue to red, corresponding to the base-pairing probability from 0 to 1 [33].

Previous research by Rogers and Joyce suggests that the substrate binds in a complementary manner near the single-stranded 3'-end of the ribozyme [16]. Given this consideration, we predicted the ligation product (not the ribozyme itself) using CentroidFold [31,32,33]. CentroidFold determined that the ligated product of R3C ligase ribozyme has a typical three-way junction structure (Figure 9a); R3C ligase ribozyme itself did not show such a three-way junction structure in CentroidFold. In the case of hammer-deleted mutants, even the ligated form of <Δ9-14,GAAA> was predicted to have a three-way junction structure, although only two possible stem-forming base pairs existed (Figure 9b).

Both <Δ9-14,GAAA,35-39> and <Δ9-14,GAAA,35-39,U34C> are composed of 50 nt, yet the ligation activity was quite different between the two (Figure 7). To focus on the differences between these two mutants, the secondary structures of P4 and P5 in each were compared using CentroidFold predictions. Although <Δ9-14,GAAA,35-39> forms a tetra-stem and a hepta-loop (Figure 9c), <Δ9-14,GAAA,35-39,U34C> forms a penta-stem (with a bulge) and a tetra-loop (Figure 9d).

## 4. Discussion

Eigen predicted that the self-replication of RNA under Darwinian selection in an enzyme-free system places a limit on nucleotide length (no more than ~100 nt) [25]. In that sense, R3C ligase ribozyme (73 nt) may have unique importance in the evolution of the putative RNA world, in spite of its slower ligation activity (0.32 min^−1^) [16]. In the present study, we succeeded in shortening the ribozyme to a size that can be accomplished by the catalytic functions of montmorillonite (~50 nt) [26].

The ligation activity for the RNA substrate was much higher than that for DNA substrate in all cases, including the deletion mutants. Furthermore, we observe similar trends in mutants’ activities when comparing DNA to RNA substrate. This finding may be attributable to the fact that the complementary RNA-RNA interaction in the single-stranded 3'-terminal region is stronger than RNA-DNA interaction [35,36,37,38], or the role of 2'-hydroxyl groups of RNA substrate.

Critical regions were located within the 9–25 base pair (bp) and 10–24 bp in the hammer stem (P3) and within the 33–50 bp and 34–49 bp in the grip stem (P5). Grip and hammer-double deletion mutants within these regions caused marked decreases in the ligation activities regardless of the substrate. Based on the results of our experiment, we choose to focus on the <Δ10-14,GAAA,35-39> mutant. We cannot deny that the spontaneous ligation reaction may occur by using pyrophosphate as a leaving group because R3C ribozyme possesses triphosphate (not monophosphate) at the 5'-terminus. Given that <Δ10-14,GAAA,35-39> and further deleted mutants behave differently from one another, we conclude that <Δ10-14,GAAA,35-39> contains the crucial structural elements needed for ligation.

The CD spectra of several mutants were similar to that of full-length R3C ribozyme (Figure 8), suggesting that even such truncated mutants possess the correct conformation required for proper activity. The slight blue or red shifts of the peaks are likely due to the large difference in the components between full-length R3C ribozyme and the other mutants rather than a consequence of single- and double-stranded transitions in the RNA structures [39].

The results of the ligation activity and the structural prediction of <Δ9-14,GAAA,35-39,U34C> suggest that the stability of the grip-stem is closely related to ribozyme activity. Introduction of C at position 34 could induce a Watson-Crick interaction between C34 and G49. The relative activity of this U34C variant was higher than that of the <Δ9-14,GAAA> mutant possessing the intact grip region. This result strongly suggests that the introduced Watson-Crick C34G49 pair may confer stabilization of the stem in spite of the lack of the large part of the grip region (Figure 9d), thus explaining the highly positive impact of the U34C mutation on ribozyme activity. On the other hand, hammer-deleted mutants, even in the case of only two possible base pairs, could form the stem structure, judging from our secondary structure prediction (Figure 9b).

The three-dimensional structure of the catalytic core of an RNA-polymerase ribozyme, which is derived from the class I ligase ribozyme, takes on a tripod structure; the ligation junction is located at the three converged regions. Two A-minor triads contribute to form the tripod scaffold, and a cytosine and two phosphates are in close proximity with the ligation junction [17,18].

In the case of L1 ligase ribozyme, the catalytic core region was adjacent to the three-helix junction (Stem A, B, C) [24]. The 71-nt L1X6c ribozyme, which was optimized for crystallization, also showed a three-stranded junction structure; stems A and B stack coaxially, and stem C branches away perpendicularly from the three-strand junction. Furthermore, stem A is formed by the alignment of the 3'-end of the substrate, with the 5'-end of the ribozyme at the ligation junction, where a non-Watson-Crick, absolutely conserved G:A pair exists with two G:U pairs on either side [24]. In the case of R3C ribozyme, the ligation junction similarly contains a G:A pair, and we detected the abolishment of the activity by replacing the G:A pair with a Watson-Crick G:C pair (data not shown).

The three-stranded junction structure is commonly observed in both class I and in L1 ligase ribozymes. Our results with respect to ligation by R3C deletion mutants similarly suggest that the R3C ribozyme requires a pseudo three-stranded junction structure close to the active site. Phosphate oxygens may coordinate the Mg^2+^ cofactor, known to be needed for facilitating the reaction as shown both in class I and in L1 ligase ribozymes. A network of hydrogen bonds throughout the three-stranded junction region may stabilize both the leaving group and the transition-state geometry. It may also be that the three-stranded junction acts as a scaffold to enable the formation of such a network.

## 5. Conclusions

Despite the plausibility of the RNA world hypothesis, experimental and theoretical support for it has been insufficient at best. Ligase ribozymes may represent vestiges of the RNA world in some way, but modern scientific characterization of ribozymes has not proceeded from a fully evolutionary point of view. In the present report, we attempted to modify an artificial ligase ribozyme, R3C (73 nucleotides), by reducing the number of nucleotides in the folded ribozyme while maintaining maximum catalytic efficiency. The final shortened ribozyme we obtained comprised ~50 nucleotides, which is comparable to the estimated length of prebiotically synthesized RNA. Our findings suggest that it is indeed possible to obtain ribozymes with autocatalytic function by modifying the sequence lengths to those achievable in prebiotic conditions.
